# Modelling synaptic tau and Aβ pathology in human organotypic brain slice cultures

**DOI:** 10.1002/alz.086462

**Published:** 2025-01-03

**Authors:** Robert I. McGeachan

**Affiliations:** ^1^ Centre for Discovery Brain Sciences at the University of Edinburgh, Edinburgh, Scotland United Kingdom

## Abstract

**Background:**

Maintaining synaptic health is essential for normal neurological function, yet neurodegenerative diseases like Alzheimer’s disease and Progressive Supranuclear Palsy (PSP) exhibit synaptic loss. In these conditions, synaptic loss precedes neuronal degeneration, and the degree of synaptic loss correlates closely with the severity of clinical symptoms. Both Aβ, which accumulates in amyloid plaques in AD, and tau protein which accumulates intracellularly in tauopathies, including AD and PSP, accumulate within synaptic terminals. In model systems, the accumulation of Aβ and tau in synapses has been linked to synaptic dysfunction, synaptic loss, and the spread of pathology trans‐synaptically. However, there is currently a lack of data studying the synaptotoxic effects of these pathologic proteins in live human synapses.

**Method:**

To address this, we usenovel human organotypic brain slice cultures derived from neurosurgical peritumoral access tissue. Employing a powerful repeated measures design to account for donor tissue variability, slices from each case were cultured with either medium, soluble protein extract (sp‐extract) from PSP (n = 6) or AD (n = 5) brain, or sp‐extracts immunodepleted for tau or Aβ respectively.

**Result:**

Using high‐resolution array tomography, and linear mixed effect modelling of the data, our findings reveal that exogenous PSP‐derived tau (*p* = 0.002) and AD‐derived Aβ (*p* = 0.004) are taken up into living human post‐synapses, and within 72 hours AD‐derived Aβ causes a loss of synaptophysin puncta (*p* = 0.002). Our data further suggests that oligomeric tau has a greater propensity for synaptic uptake than phospho‐tau Ser202, Thr205 (AT8). Interestingly, PSP‐derived tau induces an astrogliosis (*p* = 0.001) and an associated increase in synaptic engulfment (*p* < 0.001) in our slice culture model.

**Conclusion:**

Human organotypic brain slice cultures can be used a valuable tool to study the effects of disease derived tau and Aβ on human synapses.

